# The Impact of Social Media on the Mental Health of Adolescents and Young Adults: A Systematic Review

**DOI:** 10.7759/cureus.42990

**Published:** 2023-08-05

**Authors:** Abderrahman M Khalaf, Abdullah A Alubied, Ahmed M Khalaf, Abdallah A Rifaey

**Affiliations:** 1 Psychiatry Department, Saudi Commission for Health Specialties, Ministry of Health, Riyadh, SAU; 2 College of Medicine, Imam Mohammad Ibn Saud Islamic University, Riyadh, SAU; 3 College of Medicine, Almaarefa University, Riyadh, SAU

**Keywords:** systematic review, young adults, adolescents, mental health, social media

## Abstract

Adolescents increasingly find it difficult to picture their lives without social media. Practitioners need to be able to assess risk, and social media may be a new component to consider. Although there is limited empirical evidence to support the claim, the perception of the link between social media and mental health is heavily influenced by teenage and professional perspectives. Privacy concerns, cyberbullying, and bad effects on schooling and mental health are all risks associated with this population's usage of social media. However, ethical social media use can expand opportunities for connection and conversation, as well as boost self-esteem, promote health, and gain access to critical medical information. Despite mounting evidence of social media's negative effects on adolescent mental health, there is still a scarcity of empirical research on how teens comprehend social media, particularly as a body of wisdom, or how they might employ wider modern media discourses to express themselves. Youth use cell phones and other forms of media in large numbers, resulting in chronic sleep loss, which has a negative influence on cognitive ability, school performance, and socio-emotional functioning. According to data from several cross-sectional, longitudinal, and empirical research, smartphone and social media use among teenagers relates to an increase in mental distress, self-harming behaviors, and suicidality. Clinicians can work with young people and their families to reduce the hazards of social media and smartphone usage by using open, nonjudgmental, and developmentally appropriate tactics, including education and practical problem-solving.

## Introduction and background

Humans are naturally social species that depend on the companionship of others to thrive in life. Thus, while being socially linked with others helps alleviate stress, worry, and melancholy, a lack of social connection can pose major threats to one's mental health [1]. Over the past 10 years, the rapid emergence of social networking sites like Facebook, Twitter, Instagram, and others has led to some significant changes in how people connect and communicate (Table 1). Over one billion people are currently active users of Facebook, the largest social networking website, and it is anticipated that this number will grow significantly over time, especially in developing countries. Facebook is used for both personal and professional interaction, and its deployment has had a number of positive effects on connectivity, idea sharing, and online learning [2]. Furthermore, the number of social media users globally in 2019 was 3.484 billion, a 9% increase year on year [3].

**Table 1:** List of social media applications available on the internet

Social media applications	Examples
Social networks	Facebook, Twitter, Instagram, Snapchat
Media sharing	WhatsApp, Instagram, YouTube, Snapchat, TikTok
Messengers	Facebook Messenger, WhatsApp, Telegram, Viber, iMessage
Blogging platforms	WordPress, Wikipedia
Discussion forums	Reddit, Twitter
Fitness & lifestyle	Fitbit

Mental health is represented as a state of well-being in which individuals recognize their potential, successfully navigate daily challenges, perform effectively at work, and make a substantial difference in the lives of others [4]. There is currently debate over the benefits and drawbacks of social media on mental health [5]. Social networking is an important part of safeguarding our mental health. Mental health, health behavior, physical health, and mortality risk are all affected by the quantity and quality of social contacts [5].

Social media use and mental health may be related, and the displaced behavior theory could assist in clarifying why. The displaced behavior hypothesis is a psychology theory that suggests people have limited self-control and, when confronted with a challenging or stressful situation, may engage in behaviors that bring instant gratification but are not in accordance with their long-term objectives [6]. In addition, when people are unable to deal with stress in a healthy way, they may act out in ways that temporarily make them feel better but ultimately harm their long-term goals and wellness [7,8]. In the 1990s, social psychologist Roy Baumeister initially suggested the displaced behavior theory [9]. Baumeister suggested that self-control is a limited resource that can be drained over time and that when self-control resources are low, people are more likely to engage in impulsive or self-destructive conduct [9]. This can lead to a cycle of bad behaviors and outcomes, as individuals may engage in behaviors that bring short respite but eventually add to their stress and difficulties [9]. According to the hypothetical terms, those who participate in sedentary behaviors, including social media, engage in fewer opportunities for in-person social interaction, both of which have been demonstrated to be protective against mental illnesses [10]. Social theories, on the other hand, discovered that social media use influences mental health by affecting how people interpret, maintain, and interact with their social network [4].

Numerous studies on social media's effects have been conducted, and it has been proposed that prolonged use of social media sites like Facebook may be linked to negative manifestations and symptoms of depression, anxiety, and stress [11]. A distinct and important time in a person's life is adolescence. Additionally, risk factors such as family issues, bullying, and social isolation are readily available at this period, and it is crucial to preserve social and emotional growth. The growth of digital technology has affected numerous areas of adolescent lives. Nowadays, teenagers' use of social media is one of their most apparent characteristics. Being socially connected with other people is a typical phenomenon, whether at home, school, or a social gathering, and adolescents are constantly in touch with their classmates via social media accounts. Adolescents are drawn to social networking sites because they allow them to publish pictures, images, and videos on their platforms. It also allows teens to establish friends, discuss ideas, discover new interests, and try out new kinds of self-expression. Users of these platforms can freely like and comment on posts as well as share them without any restrictions. Teenagers now frequently post insulting remarks on social media platforms. Adolescents frequently engage in trolling for amusement without recognizing the potentially harmful consequences. Trolling on these platforms focuses on body shaming, individual abilities, language, and lifestyle, among other things. The effects that result from trolling might cause anxiety, depressive symptoms, stress, feelings of isolation, and suicidal thoughts. The authors explain the influence of social media on teenage well-being through a review of existing literature and provide intervention and preventative measures at the individual, family, and community levels [12].

## Review

Although there is a "generally correlated" link between teen social media use and depression, certain outcomes have been inconsistent (such as the association between time spent on social media and mental health issues), and the data quality is frequently poor [13]. Browsing social media could increase your risk of self-harm, loneliness, and empathy loss, according to a number of research studies. Other studies either concluded that there is no harm or that some people, such as those who are socially isolated or marginalized, may benefit from using social media [10]. Because of the rapid expansion of the technological landscape in recent years, social media has become increasingly important in the lives of young people. Social networking has created both enormous new challenges and interesting new opportunities. Research is beginning to indicate how specific social media interactions may impair young people's mental health [14]. Teenagers could communicate with one another on social media platforms, as well as produce, like, and share content. In most cases, these individuals are categorized as active users. On the other hand, teens can also use social media in a passive manner by "lurking" and focusing entirely on the content that is posted by others. The difference between active and passive social media usage is sometimes criticized as a false dichotomy because it does not necessarily reveal whether a certain activity is goal-oriented or indicative of procrastination [15]. However, the text provides no justification for why this distinction is wrong [16]. For instance, one definition of procrastination is engaging in conversation with other people to put off working on a task that is more important. The goal of seeing the information created by other people, as opposed to participating with those same individuals, may be to keep up with the lives of friends. One of the most important distinctions that can be made between the various sorts is whether the usage is social. When it comes to understanding and evaluating all these different applications of digital technology, there are a lot of obstacles to overcome. Combining all digital acts into a single predictor of pleasure would, from both a philosophical and an empirical one, invariably results in a reduction in accuracy [17].

Methodology

This systematic review was carried out and reported in accordance with the Preferred Reporting Items for Systematic Reviews and Meta-Analyses (PRISMA) statement and standard practices in the field. The purpose of this study was to identify studies on the influence of technology, primarily social media, on the psychosocial functioning, health, and well-being of adolescents and young adults.

The MEDLINE bibliographical database, PubMed, Google Scholar, CINAHL (Cumulative Index to Nursing and Allied Health Literature), and Scopus were searched between 1 January 2000 and 30 May 2023. Social media AND mental health AND adolescents AND young adults were included in the search strategy (impact or relation or effect or influence).

Two researchers (AK and AR) separately conducted a literature search utilizing the search method and evaluated the inclusion eligibility of the discovered papers based on their titles and abstracts. Then, the full texts of possibly admissible publications were retrieved and evaluated for inclusion. Disagreements among the researchers were resolved by debate and consensus.

The researchers included studies that examined the impact of technology, primarily social media, on the psychosocial functioning, health, and well-being of adolescents and young adults. We only considered English publications, reviews, longitudinal surveys, and cross-sectional studies. We excluded studies that were not written in English, were not comparative, were case reports, did not report the results of interest, or did not list the authors' names. We also found additional articles by looking at the reference lists of the retrieved articles.

Using a uniform form, the two researchers (AK and AA) extracted the data individually and independently. The extracted data include the author, publication year, study design, sample size and age range, outcome measures, and the most important findings or conclusions.

A narrative synthesis of the findings was used to analyze the data, which required summarizing and presenting the results of the included research in a logical and intelligible manner. Each study's key findings or conclusions were summarized in a table.

Results

Study Selection

**Figure 1:**
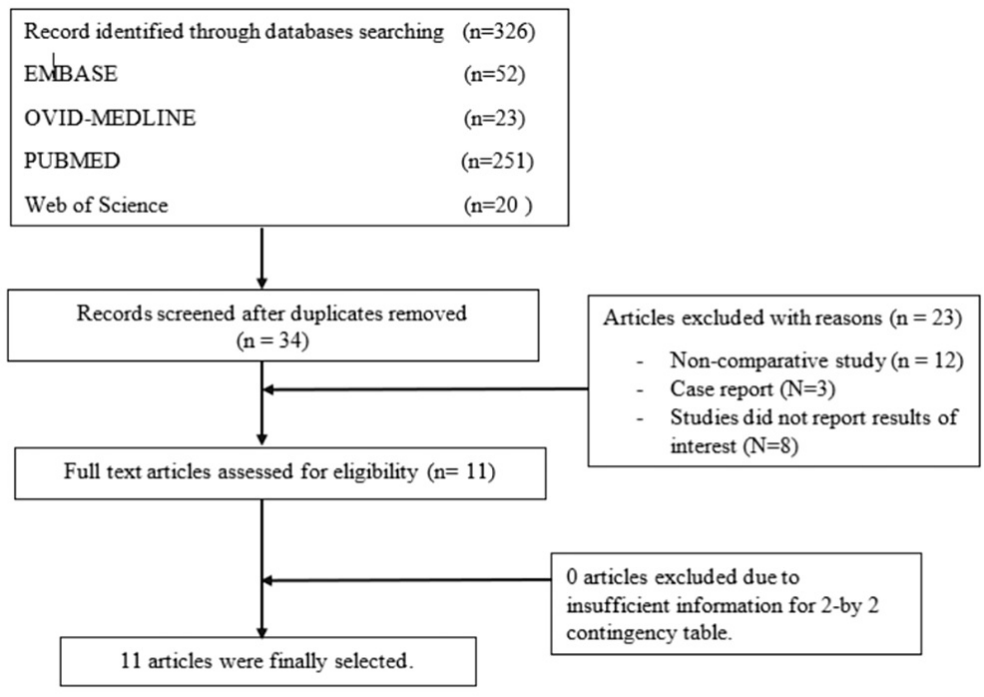
The PRISMA figure showing the steps to choose the studies for systematic review PRISMA: Preferred Reporting Items for Systematic Reviews and Meta-Analyses.

A thorough search of electronic databases, including PubMed, Embase, and Cochrane Library, was done from 1 January 2000 to 20 May 2023. Initial research revealed 326 potentially relevant studies. After deleting duplicates and screening titles and abstracts, the eligibility of 34 full-text publications was evaluated. A total of 23 papers were removed for a variety of reasons, including non-comparative studies, case reports, and studies that did not report results of interest (Figure 1).

This systematic review identified 11 studies that examined the connection between social media use and depression symptoms in children and adolescents. The research demonstrated a modest but statistically significant association between social media use and depression symptoms. However, this relationship's causality is unclear, and additional study is required to construct explanatory models and hypotheses for inferential studies [18].

Additional research studied the effects of technology on the psychosocial functioning, health, and well-being of adolescents and young adults. Higher levels of social media usage were connected with worse mental health outcomes [19], and higher levels of social media use were associated with an increased risk of internalizing and externalizing difficulties among adolescents, especially females [20]. The use of social media was also connected with body image problems and disordered eating, especially among young women [21], and social media may be a risk factor for alcohol consumption and associated consequences among adolescents and young adults [22].

It was discovered that cyberbullying victimization is connected with poorer mental health outcomes in teenagers, including an increased risk of sadness and anxiety [23]. The use of social media was also connected with more depressive symptoms and excessive reassurance-seeking, but also with greater popularity and perceived social support [24], as well as appearance comparisons and body image worries, especially among young women [25]. Children and adolescents' bedtime media device use was substantially related to inadequate sleep quantity, poor sleep quality, and excessive daytime drowsiness [26].

Online friends can be a significant source of social support, but in-person social support appears to provide greater protection against persecution [27]. Digital and social media use offers both benefits and risks to the health of children and adolescents, and an individualized family media use plan can help strike a balance between screen time/online time and other activities, set boundaries for accessing content, promote digital literacy, and support open family communication and consistent media use rules (Tables 2, 3) [28].

**Table 2:** Studies on the impact of technology on adolescents and young adults' psychosocial functioning, health, and well-being

Authors	Year	Study design	Sample size and age range	Outcome measures
McCrae et al. [18]	2017	Systematic review	11 empirical studies examining the relationship between social media use and depressive symptoms in children and adolescents	Correlation between social media use and depressive symptoms, with limited consensus on phenomena for investigation and causality
Przybylski et al. [19]	2020	Cross-sectional	National Survey of Children’s Health (NSCH): 50,212 primary caregivers	Psychosocial functioning and digital engagement, including a modified version of the Strengths and Difficulties Questionnaire and caregiver estimates of daily television- and device-based engagement
Riehm et al. [20]	2019	Longitudinal cohort study	Population Assessment of Tobacco and Health study: 6,595 adolescents aged 12-15 years	Internalizing and externalizing problems assessed via household interviews using audio computer-assisted self-interviewing
Holland and Tiggemann et al. [21]	2016	Systematic review	20 peer-reviewed articles on social networking sites use and body image and eating disorders	Body image and disordered eating
Moreno et al. [22]	2016	Review	Studies focused on the intersection of alcohol content and social media	Alcohol behaviors and harms associated with alcohol use
Fisher et al. [23]	2016	Systematic review and meta-analysis	239 effect sizes from 55 reports, representing responses from 257,678 adolescents	Peer cybervictimization and internalizing and externalizing problems
Nesi and Prinstein [24]	2015	Longitudinal	619 adolescents aged 14.6 years	Depressive symptoms, frequency of technology use (cell phones, Facebook, and Instagram), excessive reassurance-seeking, technology-based social comparison, and feedback-seeking, and sociometric nominations of popularity
Fardouly and Vartanian [25]	2016	Review	Correlational and experimental studies on social media usage and body image concerns among young women and men	Body image concerns and appearance comparisons
Carter et al. [26]	2016	Systematic review and meta-analysis	20 cross-sectional studies involving 125,198 children aged 6-19 years	Bedtime media device use and inadequate sleep quantity, poor sleep quality, and excessive daytime sleepiness
Ybarra et al. [27]	2015	Cross-sectional	5,542 US adolescents aged 14-19 years	Online and in-person peer victimization and sexual victimization, and the role of social support from online and in-person friends
Chassiakos et al. [28]	2016	Systematic review	Empirical research on traditional and digital media use and health outcomes in children and adolescents	Opportunities and risks of digital and social media use, including effects on sleep, attention, learning, obesity, depression, exposure to unsafe content and contacts, and privacy

**Table 3:** Main results or conclusions of studies on the impact of social media on adolescents and young adults' mental health, substance use, peer victimization, and technology-based behaviors

Authors	Main results or conclusions
McCrae et al. [18]	There is a small but statistically significant correlation between social media use and depressive symptoms in young people, but causality is not clear and further research is needed to develop explanatory models and hypotheses for inferential studies. Qualitative methods can also play an important role in understanding the mental health impact of internet use from young people's perspectives.
Przybylski et al. [19]	Higher levels of social media use were associated with poorer mental health outcomes, but this relationship was small and may be due to other factors.
Riehm et al. [20]	Greater social media use was associated with an increased risk of internalizing and externalizing problems among adolescents, particularly among females.
Holland and Tiggemann et al. [21]	Social media use is associated with body image concerns and disordered eating, particularly among young women.
Moreno et al. [22]	Social media may be a risk factor for alcohol use and associated harms among adolescents and young adults.
Fisher et al. [23]	Cyberbullying victimization is associated with poorer mental health outcomes among adolescents, including increased risk of depression and anxiety.
Nesi and Prinstein [24]	Social media use is associated with greater depressive symptoms and excessive reassurance-seeking, but also with greater popularity and perceived social support.
Fardouly and Vartanian [25]	Social media use is associated with appearance comparisons and body image concerns, particularly among young women.
Carter et al. [26]	Bedtime media device use is strongly associated with inadequate sleep quantity, poor sleep quality, and excessive daytime sleepiness in children and adolescents. An integrated approach involving teachers, healthcare providers, and parents is needed to minimize device access and use at bedtime.
Ybarra et al. [27]	Online friends can be an important source of social support, but in-person social support appears to be more protective against victimization. Online social support did not reduce the odds of any type of victimization assessed.
Chassiakos et al. [28]	Digital and social media use offers both benefits and risks to the health of children and teenagers. A healthy family media use plan that is individualized for a specific child, teenager, or family can identify an appropriate balance between screen time/online time and other activities, set boundaries for accessing content, guide displays of personal information, encourage age-appropriate critical thinking and digital literacy, and support open family communication and implementation of consistent rules about media use.

Discussion

Does Social Media Have a Positive or Negative Impact on Adolescents and Young Adults?

Adults frequently blame the media for the problems that younger generations face, conceptually bundling different behaviors and patterns of use under a single term when it comes to using media to increase acceptance or a feeling of community [29,30]. The effects of social media on mental health are complex, as different goals are served by different behaviors and different outcomes are produced by distinct patterns of use [31]. The numerous ways that people use digital technology are often disregarded by policymakers and the general public, as they are seen as "generic activities" that do not have any specific impact [32]. Given this, it is crucial to acknowledge the complex nature of the effects that digital technology has on adolescents' mental health [19]. This empirical uncertainty is made worse by the fact that there are not many documented metrics of how technology is used. Self-reports are the most commonly used method for measuring technology use, but they can be prone to inaccuracy. This is because self-reports are based on people's own perceptions of their behavior, and these perceptions can be inaccurate [33]. At best, there is simply a weak correlation between self-reported smartphone usage patterns and levels that have been objectively verified [34,35].

When all different kinds of technological use are lumped together into a single behavioral category, not only does the measurement of that category contribute to a loss of precision, but the category also contributes to a loss of precision. To obtain precision, we need to investigate the repercussions of a wide variety of applications, ideally guided by the findings of scientific research [36]. The findings of this research have frequently been difficult to interpret, with many of them suggesting that using social media may have a somewhat negative but significantly damaging impact on one's mental health [36]. There is a growing corpus of research that is attempting to provide a more in-depth understanding of the elements that influence the development of mental health, social interaction, and emotional growth in adolescents [20].

It is challenging to provide a succinct explanation of the effects that social media has on young people because it makes use of a range of different digital approaches [37,38]. To utilize and respond to social media in either an adaptive or maladaptive manner, it is crucial to first have a solid understanding of personal qualities that some children may be more likely to exhibit than others [39]. In addition to this, the specific behaviors or experiences on social media that put teenagers in danger need to be recognized.

When a previous study particularly questioned teenagers in the United States, the authors found that 31% of them believe the consequences are predominantly good, 45% believe they are neither positive nor harmful, and 24% believe they are unfavorable [21]. Teens who considered social media beneficial reported that they were able to interact with friends, learn new things, and meet individuals who shared similar interests because of it. Social media is said to enhance the possibility of (i) bullying, (ii) ignoring face-to-face contact, and (iii) obtaining incorrect beliefs about the lives of other people, according to those who believe the ramifications are serious [21]. In addition, there is the possibility of avoiding depression and suicide by recognizing the warning signs and making use of the information [40]. A common topic that comes up in this area of research is the connection that should be made between traditional risks and those that can be encountered online. The concept that the digital age and its effects are too sophisticated, rapidly shifting, or nuanced for us to fully comprehend or properly shepherd young people through is being questioned, which challenges the traditional narrative that is sent to parents [41]. The last thing that needs to be looked at is potential mediators of the link between social factors and teenage depression and suicidality (for example, gender, age, and the participation of parents) [22].

The Dangers That Come With Young Adults Utilizing Social Media

The experiences that adolescents have with their peers have a substantial impact on the onset and maintenance of psychopathology in those teenagers. Peer relationships in the world of social media can be more frequent, intense, and rapid than in real life [42]. Previous research [22] has identified a few distinct types of peer interactions that can take place online as potential risk factors for mental health. Being the target of cyberbullying, also known as cyber victimization, has been shown to relate to greater rates of self-inflicted damage, suicidal ideation, and a variety of other internalizing and externalizing issues [43]. Additionally, young people may be put in danger by the peer pressure that can be found on social networking platforms [44]. This can take the form of being rejected by peers, engaging in online fights, or being involved in drama or conflict [45]. Peer influence processes may also be amplified among teenagers who spend time online, where they have access to a wider diversity of their peers as well as content that could be damaging to them [46]. If young people are exposed to information on social media that depicts risky behavior, their likelihood of engaging in such behavior themselves (such as drinking or using other drugs) may increase [22]. It may be simple to gain access to online materials that deal with self-harm and suicide, which may result in an increase in the risk of self-harm among adolescents who are already at risk [22]. A recent study found that 14.8% of young people who were admitted to mental hospitals because they posed a risk to others or themselves had viewed internet sites that encouraged suicide in the two weeks leading up to their admission [24]. The research was conducted on young people who were referred to mental hospitals because they constituted a risk to others or themselves [24]. They prefer to publish pictures of themselves on social networking sites, which results in a steady flow of messages and pictures that are often and painstakingly modified to present people in a favorable light [24]. This influences certain young individuals, leading them to begin making unfavorable comparisons between themselves and others, whether about their achievements, their abilities, or their appearance [47,48].

There is a correlation between higher levels of social networking in comparison and depressed symptoms in adolescents, according to studies [25]. When determining how the use of technology impacts the mental health of adolescents, it is essential to consider the issue of displacement. This refers to the question of what other important activities are being replaced by time spent on social media [49]. It is a well-established fact that the circadian rhythms of children and adolescents have a substantial bearing on both their physical and mental development.

However, past studies have shown a consistent connection between using a mobile device before bed and poorer sleep quality results [50]. These results include shorter sleep lengths, decreased sleep quality, and daytime tiredness [50]. Notably, 36% of adolescents claim they wake up at least once over the course of the night to check their electronic devices, and 40% of adolescents say they use a mobile device within five minutes of going to bed [25]. Because of this, the impact of social media on the quality of sleep continues to be a substantial risk factor for subsequent mental health disorders in young people, making it an essential topic for the continuation of research in this area [44].

Most studies that have been conducted to investigate the link between using social media and experiencing depression symptoms have concentrated on how frequently and problematically people use social media [4]. Most of the research that was taken into consideration for this study found a positive and reciprocal link between the use of social media and feelings of depression and, on occasion, suicidal ideation [51,52]. Additionally, it is unknown to what extent the vulnerability of teenagers and the characteristics of substance use affect this connection [52]. It is also unknown whether other aspects of the environment, such as differences in cultural norms or the advice and support provided by parents, have any bearing on this connection [25]. Even if it is probable that moderate use relates to improved self-regulation, it is not apparent whether this is the result of intermediate users having naturally greater self-regulation [25].

Gains From Social Media

Even though most of the debate on young people and new media has centered on potential issues, the unique features of the social media ecosystem have made it feasible to support adolescent mental health in more ways than ever before [39]. Among other benefits, using social media may present opportunities for humor and entertainment, identity formation, and creative expression [53]. More mobile devices than ever before are in the hands of teenagers, and they are using social media at never-before-seen levels [27]. This may not come as a surprise given how strongly young people are drawn to digital devices and the affordances they offer, as well as their heightened craving for novelty, social acceptance, and affinity [27]. Teenagers are interacting with digital technology for longer periods of time, so it is critical to comprehend the effects of this usage and use new technologies to promote teens' mental health and well-being rather than hurt it [53]. Considering the ongoing public discussion, we should instead emphasize that digital technology is neither good nor bad in and of itself [27].

One of the most well-known benefits of social media is social connection; 81% of students say it boosts their sense of connectedness to others. Connecting with friends and family is usually cited by teenagers as the main benefit of social media, and prior research typically supports the notion that doing so improves people's well-being. Social media can be used to increase acceptance or a feeling of community by providing adolescents with opportunities to connect with others who share their interests, beliefs, and experiences [29]. Digital media has the potential to improve adolescent mental health in a variety of ways, including cutting-edge applications in medical screening, treatment, and prevention [28]. In terms of screening, past research has suggested that perusing social media pages for signs of melancholy or drug abuse may be viable. More advanced machine-learning approaches have been created to identify mental disease signs on social media, such as depression, post-traumatic stress disorder, and suicidality. Self-report measures are used in most studies currently conducted on adolescent media intake. It is impossible to draw firm conclusions on whether media use precedes and predicts negative effects on mental health because research has only been conducted once. Adults frequently blame the media for the problems that younger generations face [30]. Because they are cyclical, media panics should not just be attributed to the novel and the unknown. Teenagers' time management, worldview, and social interactions have quickly and dramatically changed as a result of technology. Social media offers a previously unheard-of opportunity to spread awareness of mental health difficulties, and social media-based health promotion programs have been tested for a range of cognitive and behavioral health conditions. Thanks to social media's instant accessibility, extensive possibilities, and ability to reach remote areas, young people with mental health issues have exciting therapy options [54]. Preliminary data indicate that youth-focused mental health mobile applications are acceptable, but further research is needed to assess their usefulness and effectiveness. Youth now face new opportunities and problems as a result of the growing significance of digital media in their life. An expanding corpus of research suggests that teenagers' use of social media may have an impact on their mental health. But more research is needed [18] considering how swiftly the digital media landscape is changing.

## Conclusions

In the digital era, people efficiently employ technology; it does not "happen" to them. Studies show that the average kid will not be harmed by using digital technology, but that does not mean there are no situations where it could. In this study, we discovered a connection between social media use and adolescent depression. Since cross-sectional research represents the majority, longitudinal studies are required. The social and personal life of young people is heavily influenced by social media. Based on incomplete and contradictory knowledge on young people and digital technology, professional organizations provide guidance to parents, educators, and institutions. If new technologies are necessary to promote social interaction or develop digital and relational (digitally mediated) skills for growing economies, policies restricting teen access to them may be ineffective. The research on the impact of social media on mental health is still in its early stages, and more research is needed before we can make definitive recommendations for parents, educators, or institutions. Reaching young people during times of need and when assistance is required is crucial for their health. The availability of various friendships and services may improve the well-being of teenagers.
